# Elevation as a proxy for mosquito-borne Zika virus transmission in the Americas

**DOI:** 10.1371/journal.pone.0178211

**Published:** 2017-05-24

**Authors:** Alexander G. Watts, Jennifer Miniota, Heather A. Joseph, Oliver J. Brady, Moritz U. G. Kraemer, Ardath W. Grills, Stephanie Morrison, Douglas H. Esposito, Adriano Nicolucci, Matthew German, Maria I. Creatore, Bradley Nelson, Michael A. Johansson, Gary Brunette, Simon I. Hay, Kamran Khan, Marty Cetron

**Affiliations:** 1 Li Ka Shing Knowledge Institute, St. Michael's Hospital, Toronto, Canada; 2 Division of Global Migration and Quarantine, National Center for Emerging and Zoonotic Infectious Diseases, Centers for Disease Control and Prevention, Atlanta, Georgia, United States of America; 3 Centre for the Mathematical Modelling of Infectious Diseases and Department of Infectious Disease Epidemiology, London School of Hygiene & Tropical Medicine, London, United Kingdom; 4 Spatial Ecology and Epidemiology Group, Department of Zoology, University of Oxford, Oxford, United Kingdom; 5 Oxford Big Data Institute, Li Ka Shing Centre for Health Information and Discovery, University of Oxford, Oxford, United Kingdom; 6 Eagle Medical Services, LLC for Division of Global Migration and Quarantine, Centers for Disease Control and Prevention, Atlanta, Georgia, United States of America; 7 Dalla Lana School of Public Health, University of Toronto, Toronto, Canada; 8 Division of Vector-Borne Diseases, Centers for Disease Control and Prevention, San Juan, Puerto Rico; 9 Institute for Health Metrics and Evaluation, University of Washington, Seattle, Washington, United States of America; 10 Department of Medicine, Division of Infectious Diseases, University of Toronto, Toronto, Canada; Instituut voor Tropische Geneeskunde, BELGIUM

## Abstract

**Introduction:**

When Zika virus (ZIKV) first began its spread from Brazil to other parts of the Americas, national-level travel notices were issued, carrying with them significant economic consequences to affected countries. Although regions of some affected countries were likely unsuitable for mosquito-borne transmission of ZIKV, the absence of high quality, timely surveillance data made it difficult to confidently demarcate infection risk at a sub-national level. In the absence of reliable data on ZIKV activity, a pragmatic approach was needed to identify subnational geographic areas where the risk of ZIKV infection via mosquitoes was expected to be negligible. To address this urgent need, we evaluated elevation as a proxy for mosquito-borne ZIKV transmission.

**Methods:**

For sixteen countries with local ZIKV transmission in the Americas, we analyzed (i) modelled occurrence of the primary vector for ZIKV, *Aedes aegypti*, (ii) human population counts, and (iii) reported historical dengue cases, specifically across 100-meter elevation levels between 1,500m and 2,500m. Specifically, we quantified land area, population size, and the number of observed dengue cases above each elevation level to identify a threshold where the predicted risks of encountering *Ae*. *aegypti* become negligible.

**Results:**

Above 1,600m, less than 1% of each country’s total land area was predicted to have *Ae*. *aegypti* occurrence. Above 1,900m, less than 1% of each country’s resident population lived in areas where *Ae*. *aegypti* was predicted to occur. Across all 16 countries, 1.1% of historical dengue cases were reported above 2,000m.

**Discussion:**

These results suggest low potential for mosquito-borne ZIKV transmission above 2,000m in the Americas. Although elevation is a crude predictor of environmental suitability for ZIKV transmission, its constancy made it a pragmatic input for policy decision-making during this public health emergency.

## Introduction

In February 2016, at the onset of the Zika virus (ZIKV) epidemic in the Americas, governments were required to balance protecting the health of their citizens travelling abroad against their obligation to minimize unnecessary disruption to international travel and trade as per the 2005 International Health Regulations [1]. For example, when previously unaffected countries report locally-acquired, mosquito-borne cases of ZIKV, the U.S. Centers for Disease Control and Prevention (CDC) designates the country as having local active transmission and publishes a travel notice on the CDC Traveler’s Health website. While sub-national travel notices are unusual, many ZIKV affected countries are popular U.S. travel destinations with millions of annual visitors. Since a national-level travel notice could have unnecessary economic consequences, the creation of a pragmatic method to identify areas at low risk of ZIKV transmission was essential. Hence, policymakers were pressed to identify areas that were ecologically unsuitable for mosquito-borne ZIKV transmission, to refine where travel notices should *not* apply.

Delineating where ZIKV risks are negligible within the Americas presents a public health policy challenge as the risk of mosquito-borne arbovirus transmission is heterogeneous [2]. Because of the high proportion of individuals without symptoms or with subclinical illness, limited access to timely laboratory diagnostics, and suboptimal surveillance infrastructure in many countries, establishment of the precise locations of ZIKV transmission is challenging [3]. In addition, the reported distribution of the primary mosquito vector, *Aedes aegypti* (*Ae*. *aegypti*) is limited because surveillance and reporting of mosquito presence and abundance is often inconsistent within and across nations [4]. Combinations of ecological factors have been used to predict local *Ae*. *aegypti* occurrence and thus allow more precise geographic risk estimates after the introduction of ZIKV [5]; however, since the life cycle of this mosquito depends on interacting environmental factors, many cities may not fulfill necessary temperature, precipitation, and vegetative conditions to support fundamental developmental requirements of *Ae*. *aegypti*.

Elevation is a potential proxy for *Ae*. *aegypti* range as it could be more readily understood and operationalized than time-dependent meteorological factors. Elevation is an appealing environmental proxy for *Ae*. *aegypti* range because it is correlated to a variety of fundamental dynamic ecological factors critical for mosquito development, especially temperature [6], within latitudes favorable for arbovirus transmission. Elevation itself has no known direct effect on virus transmission but could be used for policy decisions as a proxy for the critical dynamic factors that influence virus transmission.

Currently, the World Health Organization uses elevation as a factor to inform travelers on the risk of yellow fever virus (YFV) acquisition, excusing the recommended vaccination for travelers whose itineraries are limited to areas above 2,300m in some African and South American regions [7,8]. To inform time-sensitive policy decisions on sub-national travel notices at the onset of the ZIKV epidemic in the Americas, we sought to identify an elevation threshold where the probability of *Ae*. *aegypti* occurring and the associated predicted risk of ZIKV infection become negligible.

## Methods

To determine the probability of *Ae*. *aegypti* occurrence as a prerequisite for ZIKV transmission at varying elevations, we performed three geospatial analyses. For each 100-m elevation interval, we determined (a) the proportion of a country’s total land area where *Ae*. *aegypti* is predicted to occur above each interval, using an established species distribution modeling approach [4], (b) the size of the population living in areas where *Ae*. *aegypti* occurrence is predicted to occur above each elevation interval, derived by high resolution population maps, and (c) the number of historic human dengue cases reported above each interval, as an indicator of the longer-term extent of ZIKV transmission, assuming it follows previous patterns of expansion to dengue, a related arbovirus [9,10]. We examined the land, population, and reported case distributions of dengue to assess the feasibility of establishing an elevation threshold where the predicted risk of ZIKV transmission becomes negligible.

### Data sources

#### Elevation

We used the 30-arc-second (1 x 1 km) spatial resolution global multi-resolution terrain elevation data [11], available from the U.S. Geological Survey. Previous studies report *Ae*. *aegypti* elevation maxima in the Americas at 1,600m (moderately abundant) and 2,100m (present but rare) (Lozano-Fuentes et al. 2012). However, dengue risk has also been reported to be unlikely at elevations above 1,500m [12]. Based on these observed elevation range limits of *Ae*. *aegypti* and dengue, this analysis included countries in the Americas with local ZIKV transmission as of February 25, 2016 that have any areas at elevations of 1,500m or greater. This yielded 16 countries with local ZIKV transmission for analysis (Table 1).

**Table 1:** Elevations and population counts for 16 Zika-affected countries with elevations greater than 1,500 m [20].

Country (Mean Elevation; Maximum Elevation; Total Population)	City	Population	City Elevation
Bolivia (1,192 m; 6,542 m; 10.7M)	Santa Cruz de la Sierra	1.4M	416 m
	Cochabamba	900K	2,558 m
	La Paz	813K	3,650 m
	Sucre	225K	2,750 m
Brazil (320 m; 2,994 m; 200.4M)	Sao Paulo	10.0M	760 m
	Rio de Janeiro	6.0M	0–1,020 m
	Salvador	2.7M	8 m
Colombia (593 m; 5,700 m; 48.3M)	Bogota	7.7M	2,620 m
	Cali	2.4M	1,014 m
	Medellin	2.0M	1,538 m
Costa Rica (746 m; 3,820 m; 4.9M)	San Jose	335K	1,172 m
	Limon	63K	0 m
	San Francisco	56K	1,128 m
Dominican Republic (424 m; 3,098 m; 10.4M)	Santo Domingo	2.2M	14 m
	Santiago de los Caballeros	1.2M	175 m
	Santo Domingo Oeste	701K	58 m
Ecuador (1,117 m; 6,267 m; 15.7M)	Guayaquil	1.9M	4 m
	Quito	1.4M	2,850 m
	Cuenca	277K	2,500 m
El Salvador (442 m; 2,730 m; 6.3M)	San Salvador	526K	658 m
	Soyapango	330K	1,152 m
	Santa Ana	177K	665 m
Guatemala (759 m; 4,220 m; 15.5M)	Guatemala City	2.1 M	1,500 m
	Mixco	473K	1,714 m
	Villa Nueva	407K	1,334 m
	Quetzaltenango	120K	2,330 m
Guyana (207 m; 2,750 m; 799.0K)	Georgetown	235K	-2 m
	Linden	45K	48 m
	New Amsterdam	35K	7 m
Haiti (470 m; 2,680 m; 10.3M)	Port-au-Prince	1.2M	41 m
	Carrefour	442K	39 m
	Delmas 73	383K	194 m
Honduras (684 m; 2,870 m; 8.1M)	Tegucigalpa	851K	990 m
	San Pedro Sula	489K	83 m
	Choloma	139K	41 m
Jamaica (18 m; 2,256 m; 2.7M)	Kingston	938K	9 m
	Spanish Town	145K	31 m
	Portmore	103K	139 m
Mexico (1,111 m; 5,636 m; 122.3M)	Mexico City	8.8M	2,250 m
	Guadalajara	1.5M	1,557 m
	Puebla	1.4M	2,160 m
	Toluca	489K	2,667 m
Nicaragua (298 m; 2,107 m; 6.1M)	Managua	973K	108 m
	Leon	145K	86 m
	Masaya	130K	239 m
Panama (360 m; 3,475 m; 3.9M)	Panama	408K	17 m
	San Miguelito	322K	57 m
	Tocumen	89K	35 m
Venezuela (450 m; 4,978 m; 30.4M)	Caracas	3.0M	887 m
	Maracaibo	2.2M	12 m
	Maracay	1.8M	448 m

#### Predicted occurrence of *Aedes aegypti*

We quantified the amount of land area with predicted *Ae*. *aegypti* occurrence using a 5 × 5 km resolution global map of the modeled distribution of *Ae*. *aegypti* [4]. This ecological niche model predicts the global distribution of *Ae*. *aegypti* by combining spatially-explicit, temperature-dependent ranges of the vector based on fundamental limits of mosquito development; geo-located and confirmed *Ae*. *aegypti* occurrence points; as well as environmental covariates (*i*.*e*., vegetation, precipitation, and urban land cover) that further explain the mosquito species distribution [4]. To increase model output specificity, we reclassified the range of probabilities of predicted *Ae*. *aegypti* occurrence (range: 0–0.99; ‘0’ being lowest and ‘0.99’ being highest) as: ‘absence’ (i.e., all values within the range: 0–0.49 reclassified to ‘0’) or ‘presence’ (i.e., all values within the range: 0.5–0.99 reclassified to ‘1’), resulting in a binary raster of ‘0’ (absence) and ‘1’ (presence). For this study, we assumed that *Ae*. *albopictus* is a less competent vector relative to *Ae*. *aegypti* for human ZIKV transmission in nature [13] as previously shown for dengue [14] and restricted our analyses to *Ae*. *aegypti*.

#### Human population

To quantify the estimated population where *Ae*. *aegypti* is predicted to occur within each elevation range, we used the LandScan (2014)^TM^ high resolution global population distribution database [15]. LandScan uses Geographic Information Systems and remote sensing technology to model average population counts over a 24-hour period for every 1x1 km area, globally.

#### Historic cases of dengue

DENV is another flavivirus transmitted by *Ae*. *aegypti* mosquitoes. We assumed that the geographic extent of ZIKV transmission would be similar to that observed for DENV, based on evidence that temperature-dependent constraints on mosquito survival and associated capability to spread arboviruses to humans is similar across these and other related flaviviruses [16]. In addition, recent studies show that viral dynamics of DENV and ZIKV in humans are similar [17]. To estimate the elevation range where dengue cases are negligible, we used a global geographic database of dengue cases between 1960–2012 (N = 8,309, [18]). This is the most comprehensive database of confirmed dengue cases with enough detailed information to carry out modelling at a sub-national scale. Cases are represented as occurrence data, linked to point or polygon locations, derived from peer-reviewed literature, case reports, or informal online sources.

### Statistical analysis

We reclassified the elevation spatial data to represent land area as vertical elevation intervals. The vertical elevation intervals were divided into three classes: (1) elevations between 0–1,000m; (2) elevations between 1,000m and 2,500m, subsequently divided into fifteen 100m ranges; and (3) elevations greater than 2,500m, to a maximum of 8,800m. This resulted in 17 elevation ranges used to estimate land area with predicted *Ae*. *aegypti* occurrence, human population counts in those areas, and historic human dengue cases. We chose to further divide the second elevation class by fifteen 100m intervals between 1,000m and 2,500m to ensure that we captured any potential elevation level that might fall between the estimated range of *Ae*. *aegypti* that has been previously observed in the Americas (*i*.*e*., between 1,700m and 2,100m [19]).

We quantified the land area where *Ae*. *aegypti* is predicted to occur within each elevation range per country. First, we multiplied the reclassified binary raster of predicted *Ae*. *aegypti* occurrence (5 x 5 km) [4] with the reclassified elevation raster to standardize the spatial resolution between the *Ae*. *aegypti* and elevation models for each 100m increment elevation interval. To evaluate an elevation interval above which *Ae*. *aegypti* is expected to occur or not occur with very rare frequency, we divided the aggregated sum of land area classified as *Ae*. *aegypti-*‘present’ above each elevation interval by the total land area of each country with local ZIKV transmission. These estimates resulted in a value for the remaining land area (%) where there is predicted *Ae*. *aegypti* occurrence above each elevation level, for each analyzed country.

To evaluate the population at risk of encountering *Ae*. *aegypti* for the 16 analyzed countries with local ZIKV transmission, we selected all LandScan pixels that intersected the binary *Ae*. *aegypti* occurrence raster within each elevation range using zonal statistics (i.e., measures of descriptive statistics for a spatial entity within a given geographic area). We then summed the estimated population within those pixels. We compared the population at risk of encountering *Ae*. *aegypti* above all elevation intervals for each analyzed country, assuming that populations residing in an area predicted to have *Ae*. *aegypti* occurrence puts them at risk of encountering *Ae*. *aegypti*.

We used historic dengue occurrence points to validate the likelihood of ZIKV transmission at varying elevations. We first selected all dengue cases (n = 2,950) reported from the 16 analyzed countries. We removed 248 occurrence points (8.4% of total cases) where the location of occurrence could not be confirmed at a city or point level of geographic resolution (e.g. those that were reported only at state or provincial level) due to high variability in elevation (and therefore uncertainty of the specific location of DENV infection, within those states or provinces). We then counted the remaining geo-positioned dengue cases (n = 2,702) that were located within each elevation interval. Finally, we calculated the proportion of all reported human dengue cases reported above each elevation interval within each of the 16 analyzed countries.

Using this elevation-specific modeled *Ae*. *aegypti* distribution map, population counts, and geo-located dengue cases for the 16 countries with local ZIKV transmission, we defined three criteria for choosing an elevation threshold where the risk of ZIKV exposure by *Ae*. *aegypti* mosquitoes becomes negligible: (a) where the average proportion of land area above a given elevation with predicted *Ae*. *aegypti* occurrence is approximately 1% or less of total land area of the selected country or territory; (b) the proportion of the human population living above a given elevation and where *Ae*. *aegypti* is predicted to occur is approximately 1% or less of the total population of the selected country or territory; and (c) the proportion of dengue case points reported above a given elevation is approximately 1% or less of the total number of reported dengue case points for each selected country or territory. We chose 1% as the criterion for rare occurrence of *Ae*. *aegypti* and associated disease risks to account for possible error in the *Ae*. *aegypti* distribution model and reporting biases in the dengue case points. To be conservative, we strictly chose the maximum elevation threshold that met any one of these three criteria.

## Results

During the initial stages of the ZIKV epidemic in the Americas (i.e. as of February 26, 2016), 16 of 36 countries with reported local ZIKV transmission were deemed to have physical geographic features at or above 1,000m. Table 1 displays the mean and maximum elevations and total population in each of the 16 countries, and the population and elevation of the most populous cities in each of these countries [20].

**Fig 1:**
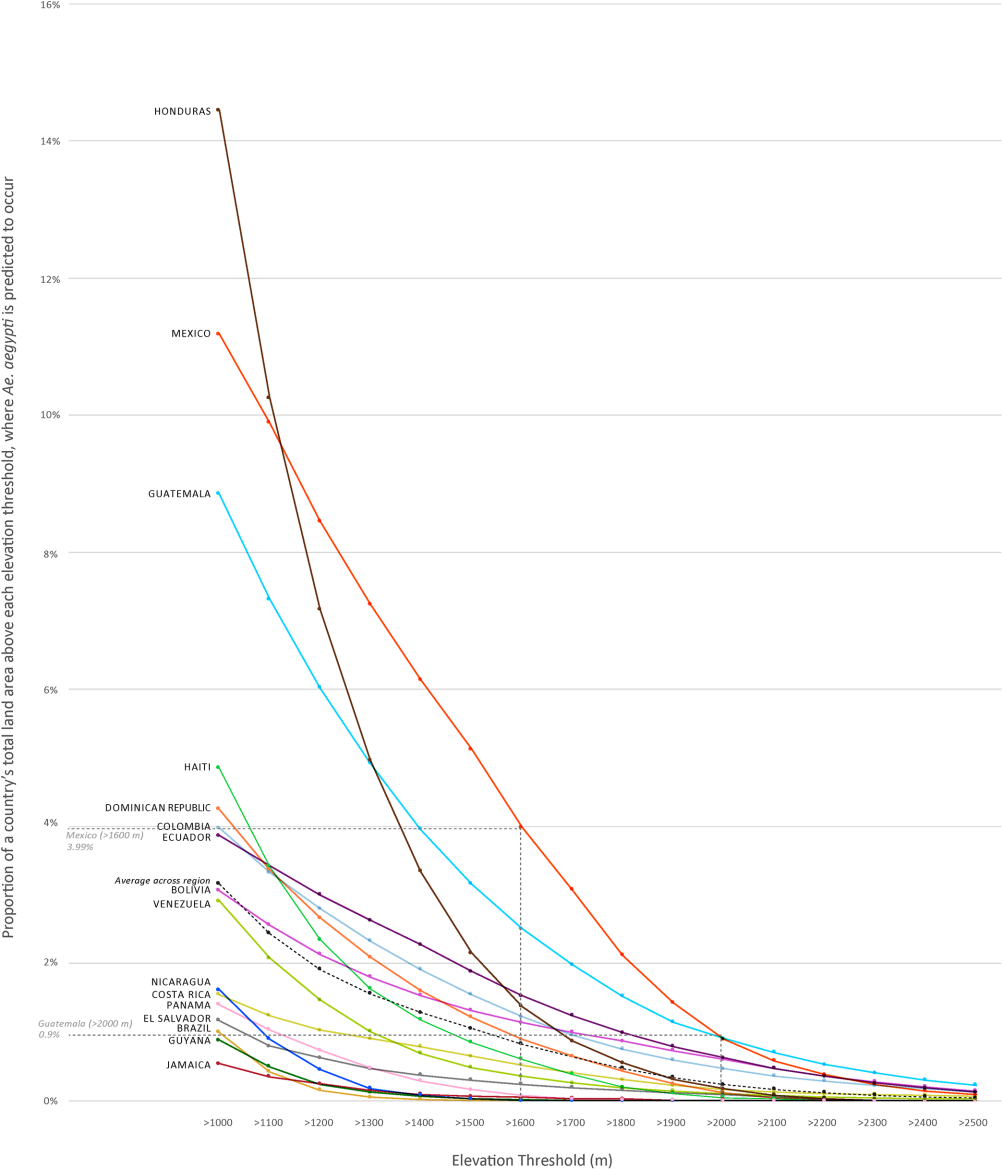
Proportion of a country’s total land area above each elevation threshold, where *Ae*. *aegypti* is predicted to occur.

At higher elevations, less land area was predicted to have *Ae*. *aegypti* (Fig 1). On average across the 16 countries, 1% or less land area above 1,600m was predicted to have *Ae*. *aegypti* (range: 0.02% in Nicaragua to 3.99% in Mexico). The three countries with the greatest proportion of land area above 1,600 m where *Ae*. *aegypti* occurrence was also predicted, were Mexico (3.99%), Guatemala (2.51%), and Ecuador (1.53%).

**Fig 2:**
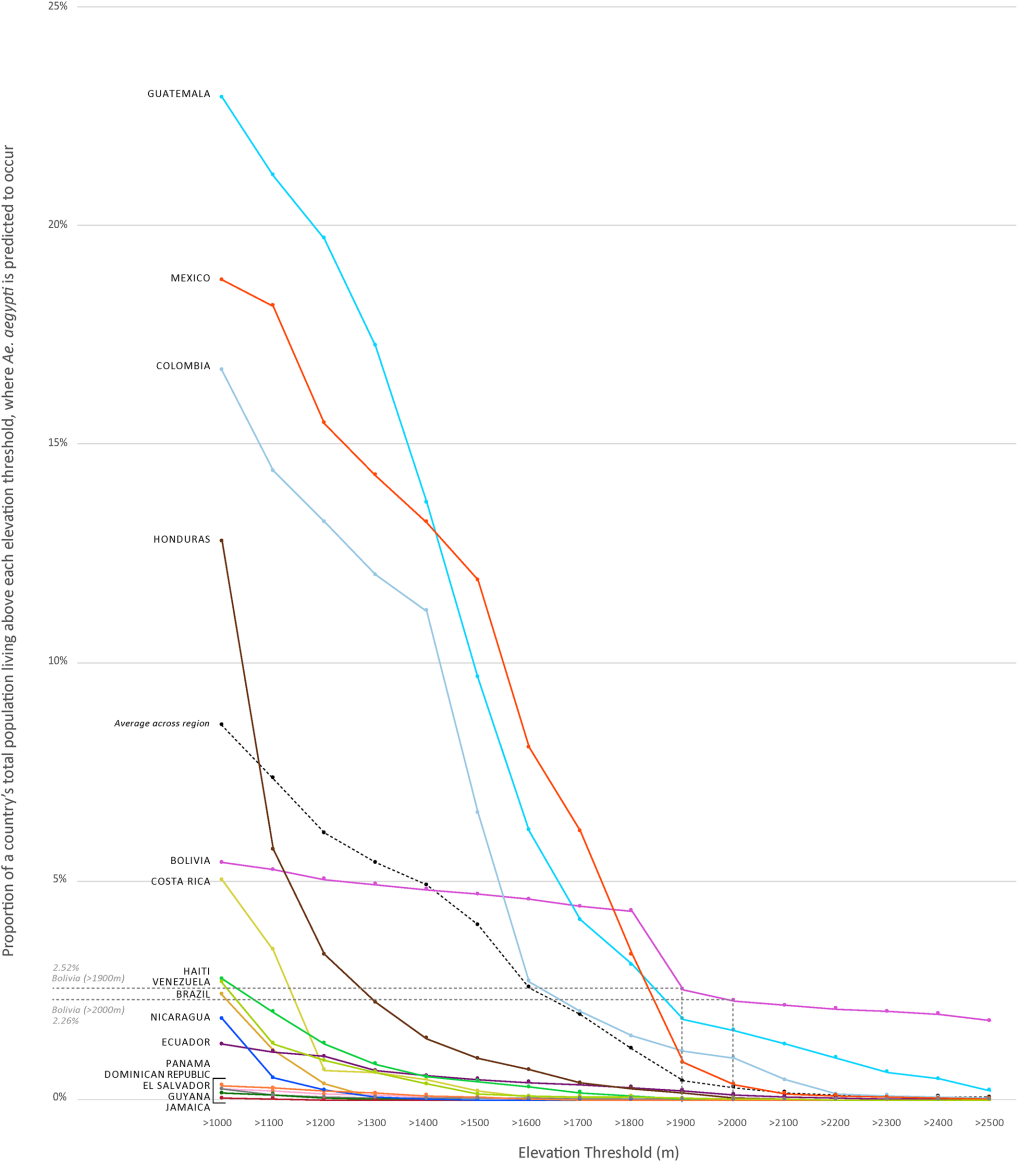
Proportion of a country’s total population living above each elevation threshold, where *Ae*. *aegypti* is predicted to occur.

At higher elevations, the predicted risk of humans encountering *Ae*. *aegypti* also decreased. On average across the 16 countries, 1% or less of the total human population living above 1,900m remained at risk of encountering *Ae*. *aegypti* (Fig 2); these values ranged from 0.00% (Brazil, Dominican Republic, Guyana, Jamaica, Nicaragua, and Panama) to 2.52% (Bolivia). Countries with the greatest proportion of population above 1,900m and predicted to have *Ae*. *aegypti* occurrence were Bolivia (2.52%); Guatemala (1.84%); and Colombia (1.11%).

On average across the 16 countries, 1% or less of all historically reported dengue cases were observed above 2,000m (Table 2); six of the 16 analyzed countries reported some dengue cases above 2,000m. Of the reported dengue cases used for this analysis [18], Colombia had the highest number located above 2,000m (11 cases; 9.6% of all cases in Colombia) followed by Bolivia (eight cases; 9.4% of all cases in Bolivia) and Mexico (six cases; 2.6% of all cases in Mexico).

**Table 2:** Geo-located human dengue virus (DENV) cases reported by Messina et al. 2014 in the 16 Zika-affected countries for all elevation intervals (m).

Countries	<1000	1,100	1,200	1,300	1,400	1,500	1,600	1,700	1,800	1,900	2,000	2,100	2,200	2,300	2,400	2,500	>2,501	Total number of reported DENV cases (1968–2012)	Total number of DENV cases reported above 2,000m	Proportion of cases reported above 2,000m to all cases within country
**Bolivia**	67	1			3	2		1	1	2		1					7	85	8	9.41%
**Brazil**	1770																	1,770	0	0.00%
**Colombia**	78	7	4	3	3	5		1	1		2	2	2	2			5	115	11	9.57%
**Costa Rica**	55						1					1						57	1	1.75%
**Dominican Republic**	37																	37	0	0.00%
**Ecuador**	48	4															2	54	2	3.70%
**El Salvador**	30																	30	0	0.00%
**Guatemala**	8					6												14	0	0.00%
**Guyana**	4																	4	0	0.00%
**Haiti**	12																	12	0	0.00%
**Honduras**	24	1	1	10		2				1								39	0	0.00%
**Jamaica**	3																	3	0	0.00%
**Mexico**	164	2	7	6	6	7	16	9	7	3		2		1	2		1	233	6	2.58%
**Nicaragua**	54	1																55	0	0.00%
**Panama**	40																	40	0	0.00%
**Venezuela**	143	6		1			1	1		1							1	154	1	0.65%
**Total**																		2,702	29	1.07%

**Fig 3:**
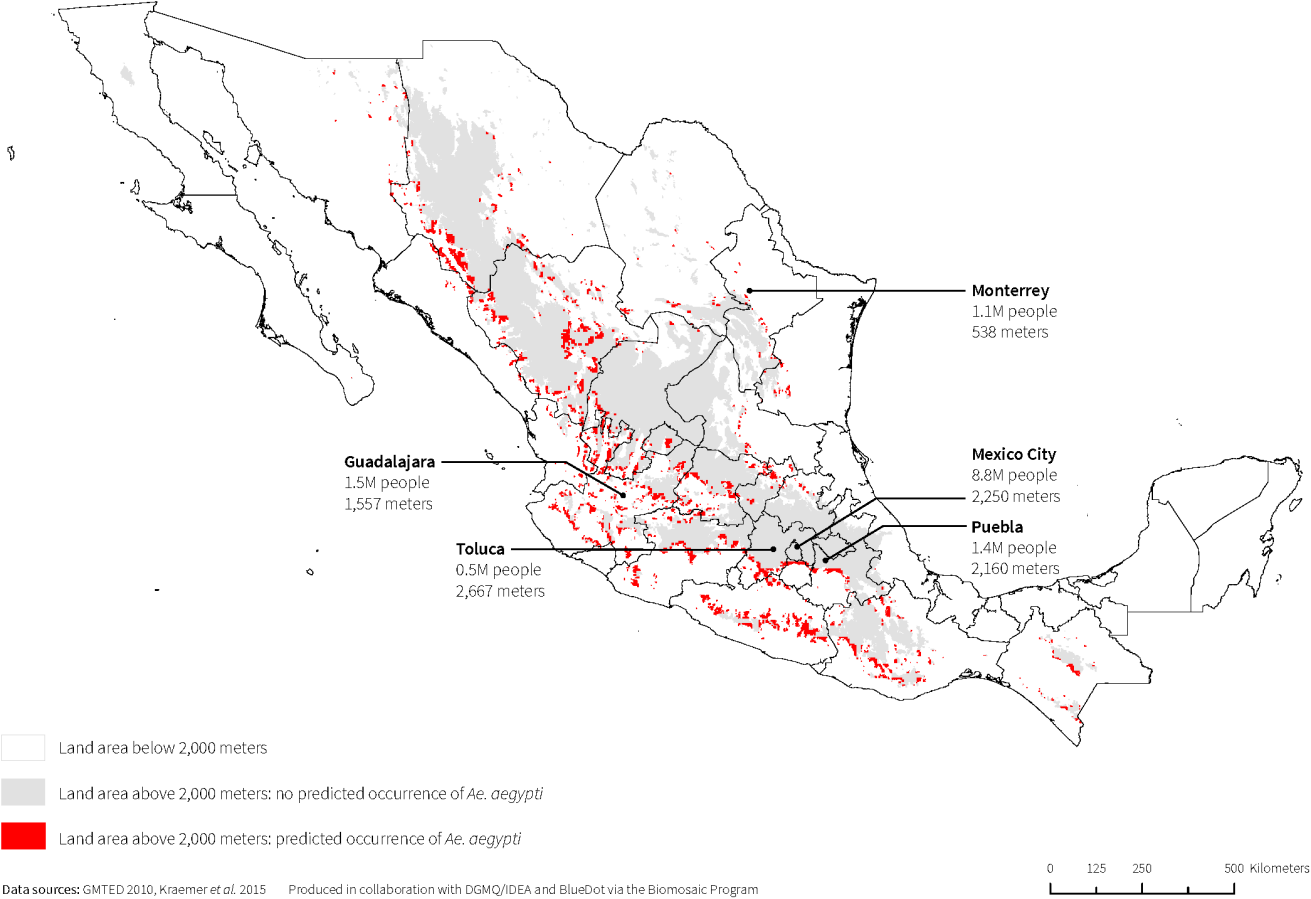
Mexico’s land area above 2,000m where *Ae*. *aegypti* is predicted to occur.

**Fig 4:**
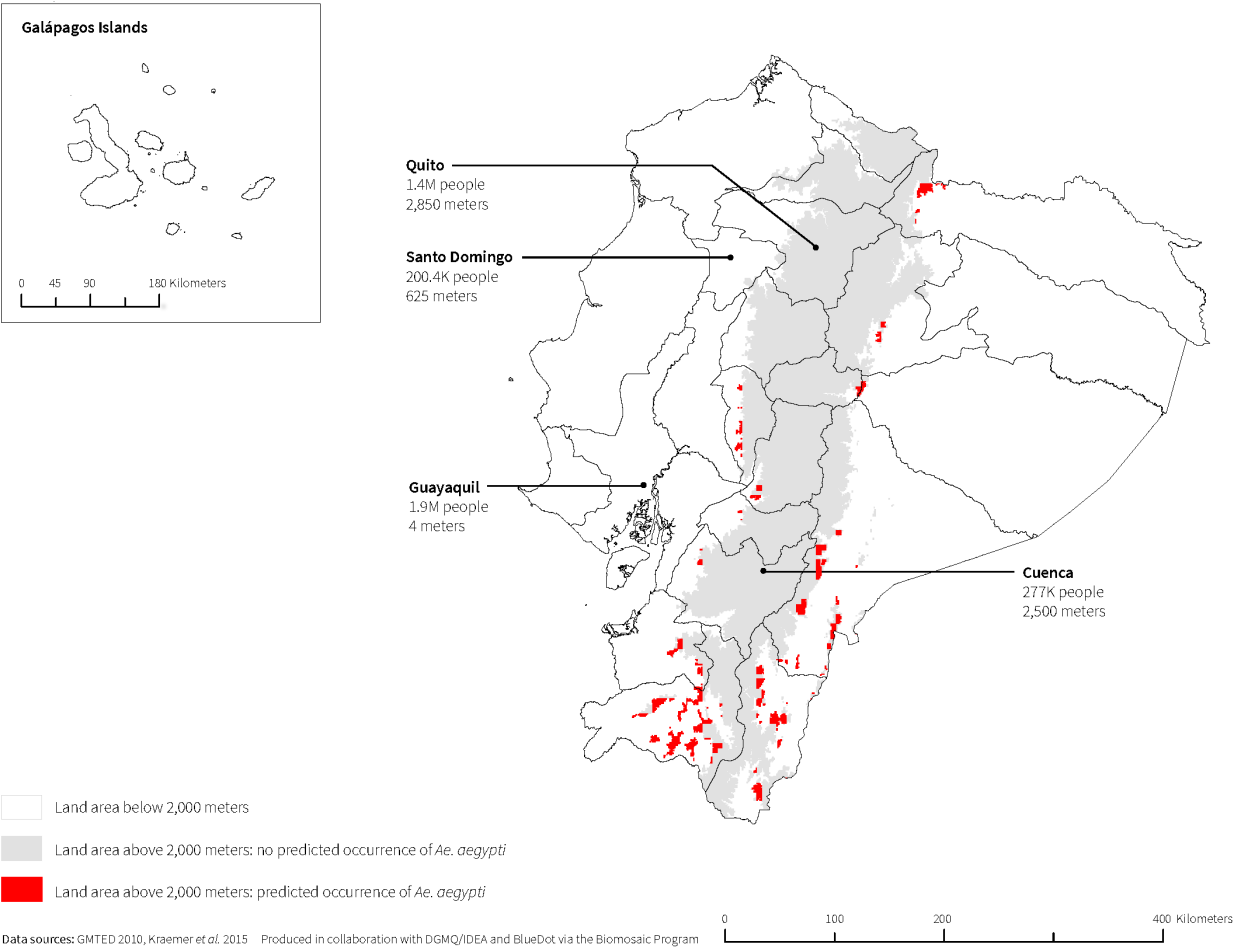
Ecuador’s land area above 2,000m where *Ae*. *aegypti* is predicted to occur.

The maximum elevation that met any one of the three threshold criteria was 2,000m; therefore, we calculated *Ae*. *aegypti* coverage and corresponding populations above 2,000m. On average across the 16 countries, above 2,000m, *Ae*. *aegypti* was predicted to occur in less than 0.25% of the total land area. Countries with the greatest proportion of land area above 2,000m predicted to have *Ae*. *aegypti* occurrence were Guatemala (0.90%); Mexico (0.89%); Ecuador (0.62%); and Bolivia (0.60%). Brazil, Guyana, Nicaragua, and Panama were predicted to have no *Ae*. *aegypti* occurrence above 2,000m. On average across the 16 countries, less than 0.28% of total population living above 2,000m was estimated to be at risk of encountering *Ae*. *aegypti*. In terms of human population distribution, countries with the largest populations living above 2,000m and in areas where *Ae*. *aegypti* was predicted to occur were Colombia (441,836; 1.0% of total Colombian population); Mexico (416,113; 0.4% of total Mexican population, Fig 3); Bolivia (240,759; 2.3% of total Bolivian population); and Guatemala (230,840; 1.6% of total Guatemalan population) (Tables 1 and 3). Conversely, Mexico City, Mexico (8.8 million); Bogota, Colombia (7.7 million); Puebla, Mexico (1.4 million); Quito, Ecuador (1.4 million, Fig 4); and Cochabamba, Bolivia (0.9 million), all above 2,000m, were not among cities predicted to have *Ae*. *aegypti* (Figs 3 and 4).

**Table 3:** Proportions of land area and populations with predicted occurrence of Ae. aegypti above 2,000 m for 16 Zika-affected countries in descending order of human population above 2,000m where Ae. aegypti is predicted to occur.

Countries with local ZIKV transmission	Proportion of total land area above 2,000m with predicted occurrence of *Ae*. *aegypti*	Proportion of total population living above 2,000m in areas with predicted *Ae*. *aegypti* occurrence	Human population living above 2,000m	Human population above 2,000m where *Ae*. *aegypti* is predicted to occur
**Colombia**	0.47%	1.0%	13,536,898	441,836
**Mexico**	0.89%	0.4%	38,206,848	416,113
**Bolivia**	0.60%	2.3%	6,487,452	240,759
**Guatemala**	0.90%	1.6%	2,903,382	230,840
**Ecuador**	0.62%	0.1%	6,013,513	19,215
**Venezuela**	0.10%	0.0%	158,409	7,292
**Honduras**	0.17%	0.0%	15,704	4,249
**El Salvador**	0.08%	0.0%	988	758
**Haiti**	0.04%	0.0%	5,210	306
**Dominican Republic**	0.12%	0.0%	6,102	237
**Costa Rica**	0.17%	0.0%	46,794	200
**Brazil**	0.00%	0.0%	374	101
**Guyana**	0.00%	0.0%	8,593	18
**Panama**	0.00%	0.0%	15	7
**Jamaica**	0.01%	0.0%	13	0
**Nicaragua**	0.00%	0.0%	0	0

## Discussion

Our analysis demonstrated that elevation is a crude yet practical proxy for the presence of *Ae*. *aegypti*, and consequently the risk of acquiring Zika virus infection, in the Americas. We identified that, above 1,600m and 1,900m, the predicted occurrence of *Ae*. *aegypti* and associated human risks of encountering *Ae*. *aegypti* were greatly diminished, respectively. We further demonstrated that human cases of DENV were rarely reported above 2,000m in this region of the world. Hence, using the maximum elevation that met all three of our threshold criteria, our analysis suggests that above 2,000m in the Americas environmental conditions are poorly suited for the transmission of ZIKV via *Ae*. *aegypti*.

While a single static factor such as elevation does not capture all dynamic processes that influence the spatial and temporal extent of mosquito-borne disease risks [21], we believe it is a pragmatic proxy for *Ae*. *aegypti* range because it is correlated to a variety of fundamental dynamic ecological factors critical for mosquito development and transmission of disease. For example, our results rely heavily on model outputs of the global predicted occurrence of *Ae*. *aegypti* [4] which uses a temperature-driven suitability filter defining the fundamental limits of *Ae*. *aegypti*, in addition to other model predictors of *Ae*. *aegypti* species range [5,22]. Vectorial capacity, defined as the average rate at which potentially infective mosquito bites arise following the introduction of a single infectious host, is highly influenced by extrinsic environmental factors [23,24]. Temperature and humidity highly influence the probability of daily survival and gonotrophic period for successful oviposition of eggs [6,25]. Because *Ae*. *aegypti* suitability is heavily temperature-dependent [4], it is likely that above 2,000m the temperature-dependent capacity of *Ae*. *aegypti* mosquitoes to survive long enough to transmit ZIKV to humans is limited.

Topography, notably elevation, has previously been used to identify vertical thresholds in mosquito-borne pathogen transmission [19,26,27]. Thermal constraints on adult mosquitoes have precluded the successful development and occurrence of *Ae*. *aegypti* in some high-elevation regions in south-east Australia [28]. Estimates of elevation maxima of *Ae*. *aegypti* in Mexico report that *Ae*. *aegypti* was commonly observed up to 1,700m and present but rare from 1,700m to 2,130m [19]. In Peru, researchers reported small dengue outbreaks that occurred at elevations up to approximately 1,500m [29]. However, in Colombia, *Aedes* spp. mosquitoes have been reported to survive a life-cycle indoors at elevations as great as 2,200m [30]. Together, these findings support the assertion that above 2,000m sustained *Ae*. *aegypti* populations are only partially supported by human dwellings above this elevation level. With increased surveillance capacity over the tropical Americas, especially in well-connected cities at high elevations such as La Paz (Bolivia), Quito (Ecuador), and Bogota (Colombia), our model would be improved by re-evaluating the elevation thresholds above which ongoing observed occurrences of both *Ae*. *aegypti* and confirmed cases of DENV and ZIKV are reported. Additionally, modeling the predicted occurrence of ZIKV, parameterized by improved measures of the temperature-dependent constraints on ZIKV transmission to humans, could help identify rare locations above 2,000m at risk of ZIKV transmission. While the purpose of this analysis was to provide advice more generally regarding areas more or less likely to be at risk of ZIKV transmission, travelers should always consider local conditions prior to travel as exceptions do occur.

Our study has several limitations. In the *Ae*. *aegypti* model [4], all environmental covariates used were annual summaries of seasonal conditions. As a result, our analysis does not account for seasonality variability in suitability for ZIKV transmission. Many areas within the 1,600–1,900m elevation range are likely to experience only limited windows of time during which *Ae*. *aegypti* might persist. Thus, especially in the winter time, 2,000m might be an overly conservative elevation threshold beyond which ZIKV transmission might occur [31].

Furthermore, we assumed that DENV occurrences reflect potential ZIKV occurrences at similar elevations. This assumption is limited given that specific temperature-dependent constraints on DENV transmission to humans closely resemble–but are not perfectly aligned–to those conditions essential for ZIKV transmission to humans [14]. However, regional geographic distributions and recent spread of ZIKV has followed that of DENV [32,33], and such strong similarities in the transmission characteristics between these arboviruses support our assumption without confirmed ZIKV cases in the study region at the time of investigation.

Our analysis of dengue cases in the 16 countries was performed on a historic sample dataset of geo-positioned reported DENV occurrences. This dataset is limited to observations sampled in 1960–2012 and therefore does not capture cases reported after 2012 (including cases related to high incidence of DENV in the Americas during 2015 [34]). Given more updated DENV occurrences, our elevation threshold could potentially increase depending on the location of the confirmed cases. In the available historical dataset, many cases of dengue were reported in our study region but it is likely that some cases were left unreported in particular locations due to lack of reporting or laboratory infrastructure [18]. Additionally, reporting biases in the dengue dataset limit the true spatial location of encounter by a human host with the DENV-infected mosquito vector. Finally, recent evidence suggests that ZIKV geographic range might be more restricted than that of DENV [35].

This study focused on potential ZIKV transmission by the primary vector *Ae*. *aegypti*, but did not consider transmission by other *Aedes* mosquito species, such as *Ae*. *albopictus*. Historical observations of ZIKV in *Ae*. *albopictus* mosquitoes [36] and more recent PCR-detected ZIKV infection of *Ae*. *albopictus* mosquitoes captured in the environment in Mexico [37], suggests that this species may be a competent vector for ZIKV transmission. However, *Ae*. *albopictus* is often considered a less effective vector in ZIKV transmission because *Ae*. *aegypti* feeds on human hosts more frequently than *Ae*. *albopictus* mosquitoes [38,39] and is generally more susceptible to ZIKV than *Ae*. *albopictus* [9,25]. In the case of dengue transmission, *Ae*. *albopictus* has been shown to have a limited role in population-level transmission even in highly endemic settings [14]. Therefore, while *Ae*. *albopictus* may be tolerant to cooler temperatures at higher elevations relative to *Ae*. *aegypti* and may transmit ZIKV at those elevations [6,25], it is unlikely that the potential impact of *Ae*. *albopictus* on ZIKV transmission was more critical than *Ae*. *aegypti*, especially at higher elevations. The extent of the 2015–2016 Zika outbreak appears to support these decisions as no transmission was reported in the USA in areas where *Ae*. *albopictus* exists but did occur where *Ae*. *aegypti* is known to occur.

## Conclusions

During the initial stages of the 2016 ZIKV epidemic in the Americas, difficult time-sensitive decisions were needed to balance the health risks of travelers to countries affected by ZIKV against the economic consequences of travel notices to the entirety of these countries. On March 11, 2016, these findings were applied to U.S. ZIKV-related travel notices informing travelers that the risk of infection in areas above 2,000m was negligible [40]. Since March 11, 2016, to our knowledge, no locally-acquired, mosquito-borne infections have occurred above 2,000m in any country or U.S. territory. However, prospective validation with data from human disease and vector surveillance is needed to determine if this elevation threshold continues to reflect a low risk of exposure to ZIKV. While dynamic meteorological data might have offered additional spatio-temporal precision in assessing the occurrence of *Ae*. *aegypti*, and consequently the sub-national risk of acquiring ZIKV infection in the Americas, we found elevation to be a pragmatic proxy to inform policy and may be readily understood by travelers.
